# The research landscape of ferroptosis in the brain: A bibliometric analysis

**DOI:** 10.3389/fphar.2022.1014550

**Published:** 2022-10-18

**Authors:** Mengrong Miao, Yaqian Han, Yangyang Wang, Yitian Yang, Ruilou Zhu, Mingyang Sun, Jiaqiang Zhang

**Affiliations:** Department of Anesthesiology and Perioperative Medicine, People’s Hospital of Zhengzhou University, Henan Provincial People’s Hospital, People’s Hospital of Henan University, Zhengzhou, Henan, China

**Keywords:** ferroptosis, brain, bibliometric analysis, knowledge map, VOSviewer, CiteSpace

## Abstract

**Background:** Ferroptosis is a newly proposed concept of programmed cell death and has been widely studied in many diseases during the past decade. However, a bibliometric study that concentrates on publication outputs and research trends of ferroptosis related to the brain is lacking.

**Methods:** We retrieved publication data in the field of ferroptosis in the brain from the Web of Science Core Collection on 31 December 2021. A bibliometric analysis was performed using VOSviewer and CiteSpace software.

**Results:** Six hundred fifty-six documents focusing on ferroptosis in the brain were published from 2012 to 2021. The number of publications in this field has shown a steady increase in recent years. Most publications were from China (338) and the United States (166), while the most productive organizations were at the University of Melbourne (34) and University of Pittsburgh (23). Ashley I. Bush was the most productive author, while Scott J Dixon was the most co-cited author. The journal Free Radical Biology and Medicine published the most articles in this field, while Cell was the most cited journal. Among 656 publications, top 10 cited documents were cited at least 300 times. Among the top 20 references with the strongest citation bursts, half of the papers had a burst until 2021. The keywords analysis suggests that the top 20 keywords appeared at least 40 times. Additionally, “amyloid precursor protein” was the keyword with strongest bursts.

**Conclusion:** Research on ferroptosis in the brain will continue to be highly regarded. This study analyzed the research landscape of ferroptosis in the brain and offers a new reference for researchers in this field.

## Introduction

Ferroptosis is a new type of regulated cell death proposed by Brent R Stockwell et al. in 2012, and is usually characterized by iron accumulation and lipid peroxidation (Dixon et al., 2012). Different from other forms of cell death, such as apoptosis and necrosis, ferroptosis can be inhibited by iron chelator deferiprone (DFP) or deferoxamine (DFO), Ferrostatin-1 (Fer-1), or liproxstatin-1 (Lip-1) (Friedmann Angeli et al., 2014; Li et al., 2020; Stockwell, 2022). Additionally, changes of mitochondrial morphology under an electron microscope, such as an increase in membrane density, a decrease in crista, and rupture of the outer membrane, were usually found in ferroptosis (Tang et al., 2021). To date, ferroptosis is known to be involved in wide biological processes and has been deemed as a target for treating many diseases, such as cancer (Zhao et al., 2022), pulmonary diseases (Yang et al., 2022), ischemic organ injuries (Liu et al., 2020), and other diseases associated with the toxicity of iron and lipid peroxidation (Jiang et al., 2021).

Although the great potential and a keen interest of ferroptosis has been raised by scholars and confirmed by several bibliometric studies, these studies focused on ferroptosis without classification of the research area (Wu et al., 2021; Xiong et al., 2021; Zhang et al., 2021; Dong et al., 2022) or the field of cancer (Zhou et al., 2021; Li et al., 2022) and stroke (Chen et al., 2021) research. The research landscape of ferroptosis that has concentrated on the field of brain science is not fully understood. Hence, by using VOSviewer and CiteSpace, the current bibliometric study investigated the critical role of ferroptosis in brain research through quantifying annual publication outputs, analyzing contributions and the collaboration of countries, institutes, and authors, revealing important studies, and presenting trending topics in this field.

## Materials and methods

### Data collection

A search of the Web of Science Core Collection (WOSCC) was conducted for a bibliometric analysis of ferroptosis in the brain. The literature search and extraction were performed on 10 September 2022. The search strategy was (TS = ferroptosis or TS = ferroptotic) and (TS = brain or TS = CNS or TS = neuro* or TS = cerebral). No limitations on language were performed. The publication year was limited from 2012 to 2021. After searching, a plain text file of the full record and cited references were obtained for further analysis.

### Data analysis

The annual publication trend was analyzed using an online bibliometric tool (https://bibliometric.com/) and Excel. VOSviewer 1.6.18, which was designed by Nees Jan van Eck and Ludo Waltman (2010), was used to perform the bibliometric analysis of countries, organizations, journals, references and keywords and establish a visual network. We constructed an overlay dual-map of journals using CiteSpace 5.8 designed by Chaomei Chen (Chen, 2004). The top 20 references with the strongest citation bursts and a keyword burst analysis was also performed by CiteSpace.

## Results

### The trend in publication outputs

**FIGURE 1:**
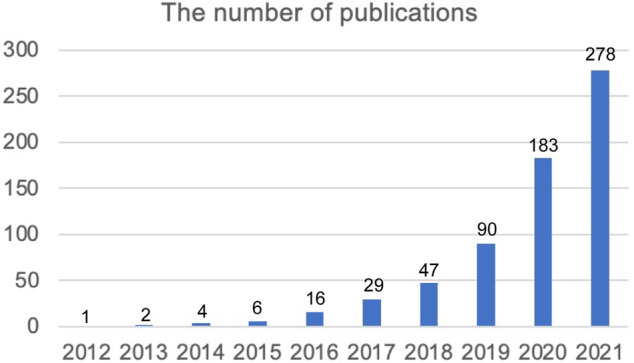
The annual article publication trend of “ferroptosis in brain” from 2012 to 2021.

After screening the WOSCC, 656 documents involving 422 articles (64.33%), 223 reviews (33.40%) and 11 editorials (1.67%) on ferroptosis in the brain were obtained (Supplementary Table S1). The annual article publication trends from 2012 to 2021 are presented in Figure 1. The publications’ Fper years from 2012 to 2015 were less than 10, but the number of publications on ferroptosis in the brain steadily increased from 2016 to 2021.

### Distribution of countries

**FIGURE 2:**
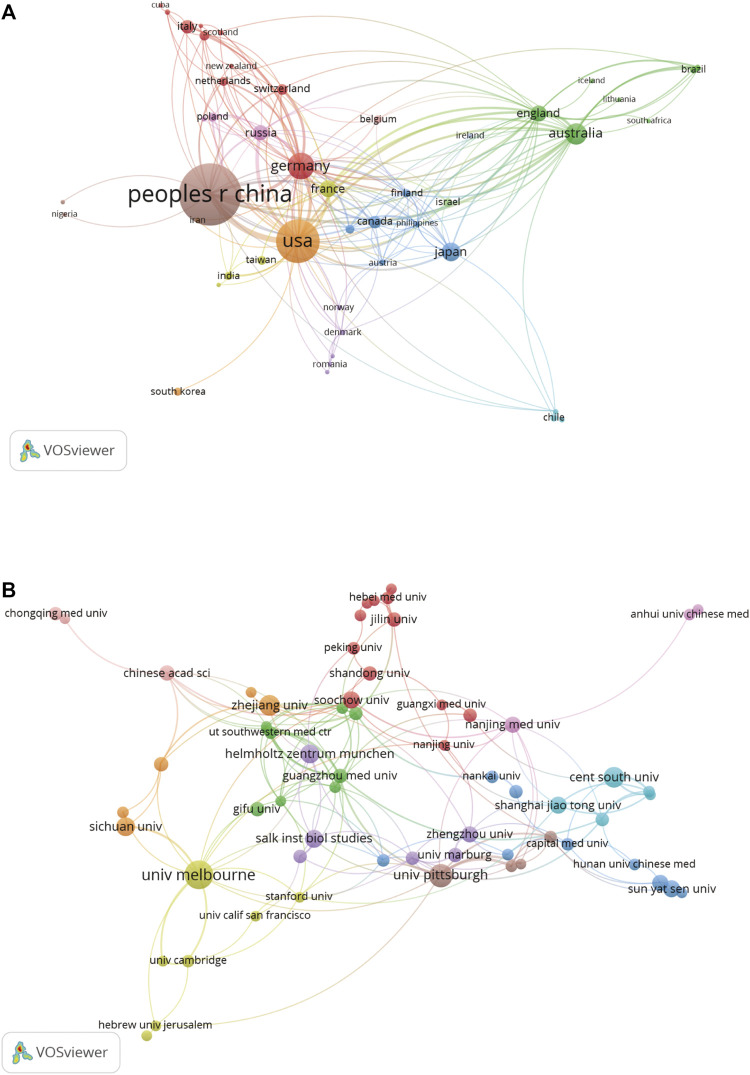
Collaborations of countries and institutions. **(A)** The collaborative relationship between different countries. **(B)** The co-author analysis of different organizations.

Visualization networks of the distribution of countries and organizations were constructed using VOSviewer 1.6. A total of 656 publications on ferroptosis in the brain were completed from 55 different countries. The co-authorship network of 51 countries is shown in Figure 2A, while the rest of the countries not shown in the figure were isolated. Each country was presented as a node in the figure, while the cluster of countries was indicated as the same color. The cooperation strength was stronger when the links between nodes were wider. We summarized the details of the publications, citations, and total link strength of 55 countries in Supplementary Table S2. The top 10 most productive countries are shown in Table 1. The top 3 countries are China (338), the United States (166), and Germany (64). Among them, the citations and total link strength of China were 11846 and 101, which were less than 18038 citations and 134 total link strengths of the United States, respectively. The citations and total link strength of Germany were 5,371 and 85, which ranked only second compared to the United States and China.

**TABLE 1:** The top 10 most productive countries.

Rank	Country	Documents	Citations	Total link strength
1	China	338	11846	101
2	United States	166	18038	134
3	Germany	64	5,371	85
4	Australia	42	4,926	57
5	Japan	33	2,953	24
6	France	27	1890	52
7	England	24	1,495	53
8	Russia	22	1,162	41
9	Italy	17	623	8
10	Canada	15	574	21

### Contributions from organizations

A total of 879 organizations completed the publication of 656 articles. Complete information regarding publications, citations, and the total link strength of 879 organizations is summarized in Supplementary Table S3. Co-authorship of 63 organizations with at least 5 publications is visualized in Figure 2B. The nodes and lines reflect the documents and cooperation of these organizations. From the visualization network, we can see that the cooperation between these organizations was active. The details of the top 10 most productive organizations are presented in Table 2. Among these organizations, six organizations came from China, two came from the United States, one came from Australia, and another was from Germany. The most productive organization was the University of Melbourne with 34 documents and a total link strength of 100. Next, the number of documents and total link strength came from the University of Pittsburgh in the United States and Central South University of China at 23 and 18 vs. 104 and 18, respectively. Data showed that the Central South University of China had less cooperation with other originations than the University of Melbourne of Australia and the University of Pittsburgh in the United States. In addition, the total citations of six organizations from China (2,510) were lower than those in Australia (4,211) and the United States (5,016). These data indicate that Australia and the United States are predominant in the field of ferroptosis in the brain.

**TABLE 2:** Top 10 most productive institutions.

	Organizations	Countries	Documents	Citations	Total link strength
1	The University of Melbourne	Australia	34	4,211	100
2	University of Pittsburgh	United States	23	4,535	104
3	Central South University	China	18	409	18
4	Zhejiang University	China	18	513	33
5	Helmholtz Zentrum Munchen	Germany	15	1,083	42
6	Sichuan University	China	15	723	21
7	Salk Institute for Biological Studies	United States	14	481	15
8	Shanghai Jiaotong University	China	13	400	22
9	Soochow University	China	12	315	19
10	Sun Yat-Sen University	China	12	150	6

### The map of co-occurrence and co-cited authors on ferroptosis in the brain

**FIGURE 3:**
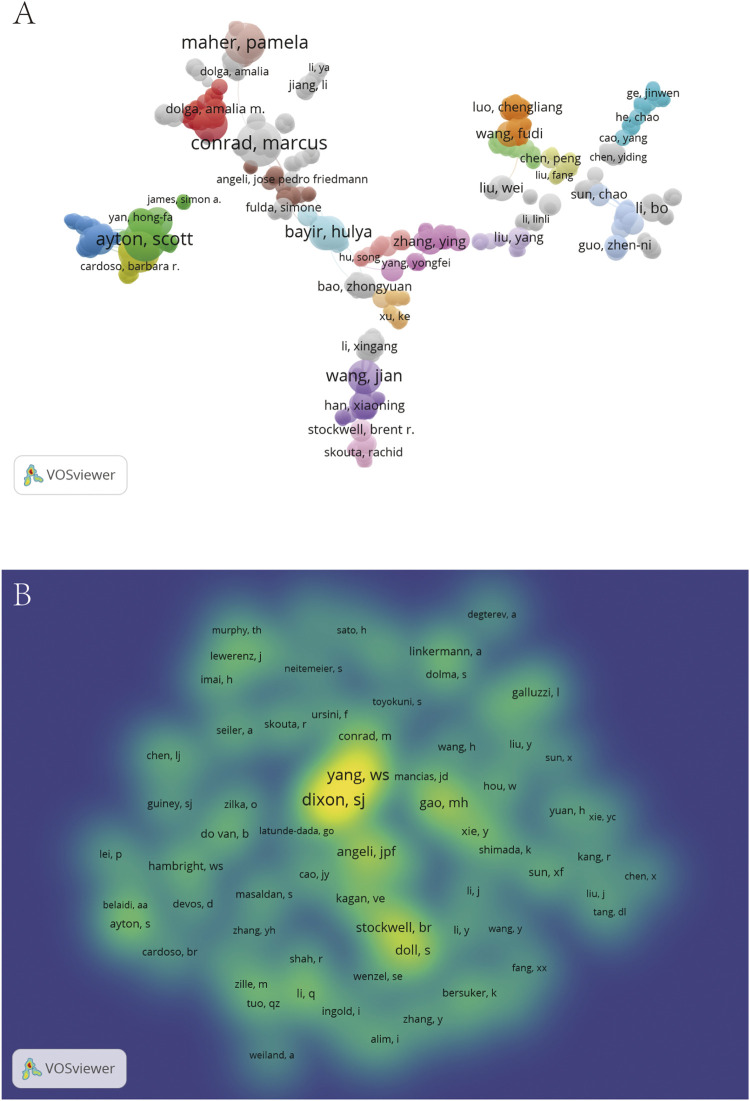
Contributions of authors on ferroptosis in the brain. **(A)** The co-occurrence network of authors. **(B)** The density map of co-cited authors.

A total of 3,759 researchers contributed to the research of ferroptosis in the brain, and documents, citations, and the total link strength of these authors are summarized in Supplementary Table S4. Among them, the co-authorship network of 1,079 authors was established using VOSviewer (Figure 3A). Close collaborations normally exist in different clusters and are presented as different colors in Figure 5A. Although Marcus Conrad, Hulya Bayir, Wang Jian, and Scott Ayton are at the center, there are also some authors isolated with other authors in the network. Data indicated a lack of cooperation among authors dedicated to the field of ferroptosis in the brain. Table 3 shows the top 10 active authors in the field of ferroptosis in the brain ranked by publication frequency. Ashley I. Bush from the University of Melbourne contributed 20 articles in this field and Marcus Conrad (Chen et al., 2021), Scott Ayton (Li et al., 2022), and Pamela Maher (Dong et al., 2022) ranked after him (Table 3).

**TABLE 3:** Top 10 authors and co-cited authors in the field of ferroptosis in the brain.

Rank	Author	Count	H-index	Co-cited author	Count	H-index
1	Ashley I. Bush	20	112	Scott J Dixon	471	31
2	Marcus Conrad	16	15	Wan Seok Yang	380	17
3	Scott Ayton	14	35	J Pedro Friedmann Angeli	306	35
4	Pamela Maher	13	56	Sebastian Doll	305	9
5	Mao Xiaoyuan	10	24	Brent R Stockwell	303	30
6	Zhou Honghao	10	46	Gao, Minghui	297	16
7	Hulya Bayir	9	35	Scott Ayton	176	35
8	Carsten Culmsee	9	57	Valerian E Kagan	173	83
9	Wang Jian	9	50	Xie Yangchun	172	22
10	Yoko Hirata	7	28	Li Qian	171	25

Among the 24072 co-cited authors, 74 authors had more than 50 co-citations (Supplementary Table S5). The density map of co-cited authors is established in Figure 3B according to color reflected co-cited frequency. The top 10 co-cited authors are listed in Table 4. From the density map, Scott J Dixon and Wan Seok Yang were the most co-cited authors. Details of the complete list of all co-cited authors that could not be shown in the map and table are summarized in Supplementary Table S5.

**TABLE 4:** Top 10 journals and co-cited journals in the field of ferroptosis in the brain.

Rank	Journal	Count	JCR (2021)	IF (2021)	Cited journal	Count	JCR (2021)	IF (2021)
1	Free Radical Biology and Medicine	24	Q1	8.101	Cell	1880	Q1	66.85
2	Frontiers in Neuroscience	21	Q2	5.152	Journal of Biological Chemistry	1,461	Q2	5.486
3	Cell Death Disease	16	Q1	9.685	Free Radical Biology and Medicine	1,365	Q1	8.101
4	Redox Biology	16	Q1	10.787	Nature	1,345	Q1	69.504
5	Frontiers in Cell and Developmental Biology	13	Q1/Q2	6.081	Proceedings of the National Academy of Sciences of the United States of America	1,239	Q1	12.779
6	Frontiers in Cellular Neuroscience	11	Q1	6.147	Cell Death and Differentiation	909	Q1	12.067
7	Cell Death and Differentiation	10	Q1	12.067	Journal of Neurochemistry	790	Q2	5.546
8	Oxidative Medicine and Cellular Longevity	10	Q2	7.31	Nature Chemical Biology	736	Q1	11.174
9	Cells	9	Q2	7.666	Redox Biology	718	Q1	10.787
10	Frontiers in Pharmacology	9	Q1	5.988	PLOS One	676	Q2	3.752

### Journals and Co-Cited journals

All 656 papers in the field of ferroptosis in the brain were published from 313 academic journals (Supplementary Table S6). The top 10 journals published 154 articles in this field and accounted for 23.5% of the number of publications (Table 4). In the top 10 journals, Free Radical Biology and Medicine published the maximum quantity of papers (Zille et al., 2017), followed by Frontiers in Neuroscience (Do Van et al., 2016) and Cell Death Disease (Chen et al., 2021). Seven of the top 10 journals were ranked in Q1 of the WoS-JCR partition.

In 3,669 co-cited sources, 110 had citations of more than 100 (Supplementary Table S7); among them, 19.5% of citations of all cited sources were from top 10 co-cited journals. Cell, Journal of Biological Chemistry, and Free Radical Biology and Medicine had the largest count of citations (Table 4).

**FIGURE 4:**
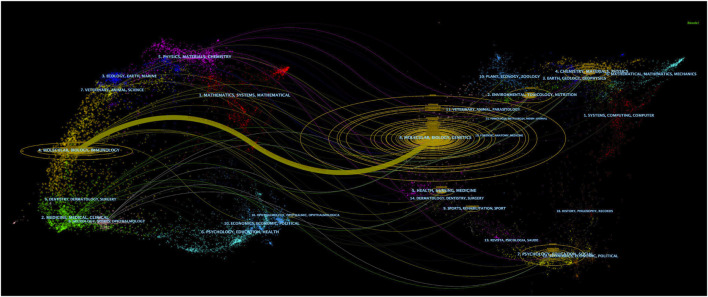
The overlay dual-map of journals on ferroptosis in the brain. The left part stands for citing journals and the right part for cited journals. The colored link between the two parts reflects the citing and cited relationship of journals.

The topic distribution of journals was represented as an overlay dual-map using CiteSpace (Figure 4). The left part of Figure 4 represents citing journals and the right part cited journals, while the link between the two parts reflects the citing and cited relationship of journals. Only one primary citation path could be found in Figure 4, which was cited from Molecular/Biology/Genetics journals to Molecular/Biology/Immunology journals.

### Top cited publications and references burst

**FIGURE 5:**
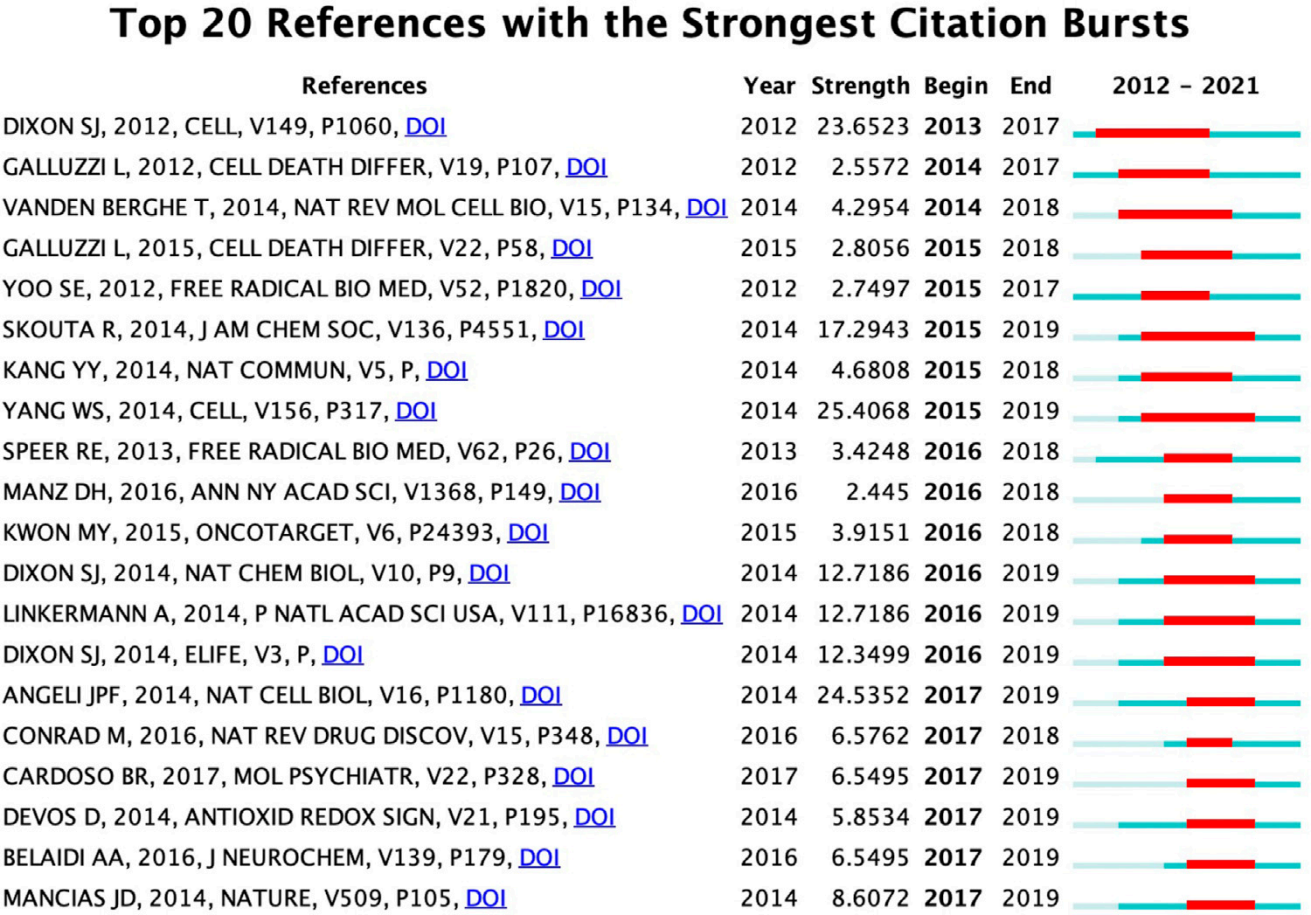
The top 20 references with the strongest citation bursts (sorted by the beginning year of the burst).

The top 10 highly cited publications in this field are found in Table 5, and all publications were cited at least 300 times. In these 10 documents, 7 were reviews (70%) and 3 were research articles (30%). An article entitled “Ferroptosis: An Iron-Dependent Form of Nonapoptotic Cell Death” published in Cell by Scott J Dixon et al. in 2012 was the most cited publication. A burst detection of references was usually used to determine references that were frequently cited in a certain period. The top 20 references with the strongest citation bursts were analyzed by CiteSpace in Figure 5. The results show that the first burst started by the paper entitled “Ferroptosis: An Iron-Dependent Form of Nonapoptotic Cell Death” published in Cell by Scott J Dixon et al. in 2012 (strength = 23.65). The strongest citation bursts occurred in an article entitled “Regulation of Ferroptotic Cancer Cell Death by GPX4” published in Cell by Wan Seok Yang et al. in 2014 (strength = 25.41), within the period from 2014 to 2019. Among these 20 references, nine papers had a burst until 2019.

**TABLE 5:** Top 10 most cited documents in the field of ferroptosis in the brain.

Rank	Title	Journal	First author	Year	Type	Citation
1	Ferroptosis: An Iron-Dependent Form of Nonapoptotic Cell Death	Cell	Scott J Dixon	2012	Article	4,283
2	Ferroptosis: A Regulated Cell Death Nexus Linking Metabolism, Redox Biology, and Disease	Cell	Brent R Stockwell	2017	Review	2,118
3	Ferroptosis: Process and Function	Cell Death and Differentiation	Xie Yangchun	2016	Review	1,228
4	Lipid Peroxidation in Cell Death	Biochemical and Biophysical Research Communications	Michael M. Gaschler	2017	Review	678
5	Dependency of a Therapy-resistant State of Cancer Cells on a Lipid Peroxidase Pathway	Nature	Vasanthi S. Viswanathan	2017	Article	633
6	Mechanisms of Ferroptosis	Cellular and Molecular Life Sciences	Cao Jennifer Yinuo	2016	Review	578
7	Mitochondria as Multifaceted Regulators of Cell Death	Nature Reviews Molecular Cell Biology	Florian J. Bock	2020	Review	518
8	Neuronal Cell Death	Molecular Psychiatry	Michael Fricker	2018	Review	394
9	NRF2 Plays a Critical Role in Mitigating Lipid Peroxidation and Ferroptosis	Redox Biology	Matthew Dodson	2019	Review	356
10	PEBP1 Wardens Ferroptosis by Enabling Lipoxygenase Generation of Lipid Death Signals	Cell	Sally E Wenzel	2017	Article	339

### Analysis of hot research topics and frontiers

**FIGURE 6:**
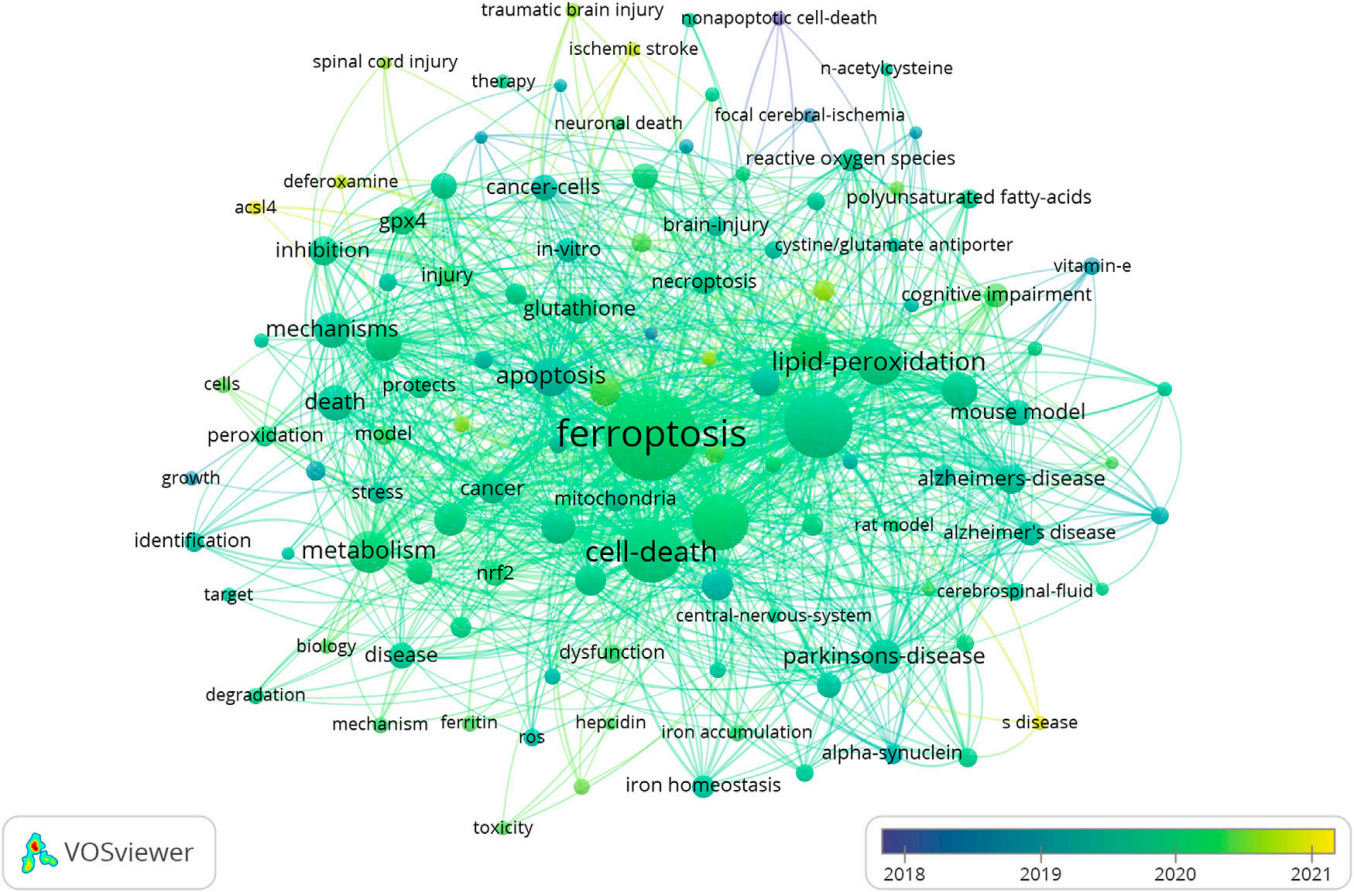
The overlay map of keywords established by VOSviewer.

The co-occurrence of all keywords involving author keywords and keywords plus of 656 publications were analyzed to investigate hot research topics in the field of ferroptosis in the brain by using VOSviewer. The co-occurrence of 2,723 keywords is summarized in Supplementary Table S8, and 117 keywords appeared at least 10 times. The overlay map of these keywords (≥10 times) was established by VOSviewer (Figure 6). The top 20 keywords appeared at least 40 times and are summarized in Table 6, and “lipid-peroxidation” and “lipid peroxidation” were merged as “lipid peroxidation,” “cell-death” and “cell death” were merged as “cell death,” “Alzheimer’s-disease” and “Alzheimer’s disease” were merged as “Alzheimer’s disease,” “Parkinson’s-disease” and “Parkinson’s disease” were merged as “Alzheimer’s disease” and “glutathione -peroxidase 4,” “gpx4” and “glutathione peroxidase 4” were merged as “glutathione peroxidase 4.” From Table 6, “ferroptosis” (437) was the most cited keyword, followed by “cell death” (247), “oxidative stress” (245), “lipid peroxidation” (198) and “iron” (163).

**TABLE 6:** Top 20 keywords related to ferroptosis in the brain.

Rank	Keyword	Occurrence	Rank	Keyword	Occurrence
1	ferroptosis	437	11	mechanisms	69
2	cell death	247	12	activation	67
3	oxidative stress	245	13	death	66
4	lipid peroxidation	198	14	brain	61
5	iron	163	15	expression	57
6	glutathione peroxidase 4	108	16	autophagy	50
7	metabolism	92	17	glutathione	48
8	Parkinson’s disease	91	18	inflammation	48
9	apoptosis	82	19	neurodegeneration	48
10	Alzheimer’s disease	74	20	cancer	47

**FIGURE 7:**
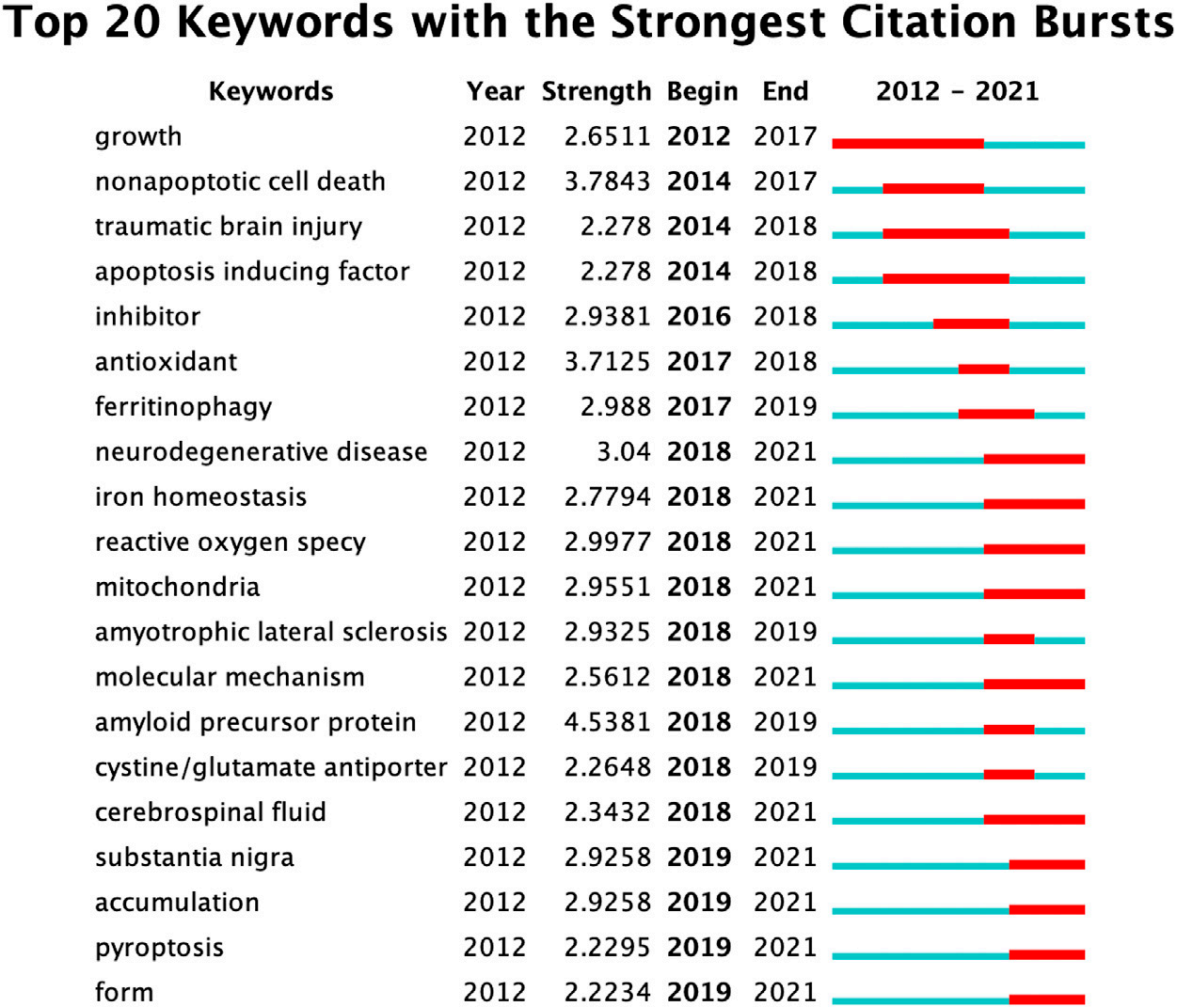
The top 20 keywords with the strongest citation bursts (sorted by the beginning year of the burst).

Keyword burst analysis was performed to find the frontiers in the current field. As shown in Figure 7, the first burst of keyword “growth” started in 2012 (strength = 2.65) followed by nonapoptotic cell death (strength = 3.78), while the strongest bursts of keywords occurred on “amyloid precursor protein” from 2018 to 2019 (strength = 4.53). In addition, half of them, such as “neurodegenerative disease,” “iron homeostasis” and “reactive oxygen species,” had bursts until 2021 (Figure 7).

## Discussion

Since ferroptosis was first defined in 2012, its role in wide biological processes and various organ damage has been investigated by researchers throughout the world. With the deepening of the research on ferroptosis, it has attracted considerable attention in the field of brain sciences. To better understand the growth trend of this topic, we performed this bibliometric analysis. Several other studies have also focused on the bibliometric analysis of ferroptosis (Chen et al., 2021; Wu et al., 2021; Xiong et al., 2021; Zhang et al., 2021; Zhou et al., 2021; Dong et al., 2022; Li et al., 2022), but these analyses examined the application of ferroptosis throughout all biological research areas without detailed division or concentrating on its role in stroke/cancer. Specifically, no analysis of its future landscape in brain research was performed. In this bibliometric analysis, we identified 656 publications on ferroptosis in the brain through a search of WOSCC from 1 January 2012 to 31 December 2021. It was surprising that more than 50% of the studies on ferroptosis in the brain were produced during the past 2 years (183 in 2021 and 278 in 2022), which indicates the explosion of this topic.

Changes in the annual number of publications in one research area usually reflect the development trend of a topic. Brent R Stockwell’s group proposed the concept of “ferroptosis” in 2012 (1), and this was first article investigating the role of ferroptosis in the brain. Although there were less than 10 papers published per year from 2012 to 2015, the annual number of publications has rapidly increased since 2016 (Figure 1). More than 50 different countries have produced evidence of ferroptosis in the brain (Supplementary Table S2), which reflects extensive global concern on this topic. Specifically, China was the most productive country, followed by the United States, but the number of citations from the United States was far greater (Table 1). These results show that China had more interest in this field than the United States, but the United States had more influence in this field. Similarly, in the top 10 productive institutes, 6 organizations were from China, but the total citation number of these organizations was still less than the University of Melbourne in Australia or the University of Pittsburgh and Salk Institute for Biological Studies in the United States (Table 2). These results suggest that China should further enhance the impact of studies, especially after the dominant tendency of the publication number in this field.

From 2012 to 2021, Free Radical Biology and Medicine and Redox Biology from the JCR 1 region published the highest number of articles in the field of ferroptosis in the brain (Table 4). In the top 10 productive journals, more than half of them were located in the JCR 1 region and all of them had an impact factor greater than 5 (Table 4). Among the top 10 most cited documents (Table 5), 3 were research articles (Dixon et al., 2012; Do Van et al., 2016; Li et al., 2017; Tuo et al., 2017; Viswanathan et al., 2017; Wenzel et al., 2017; Zille et al., 2017; Shen et al., 2018; Alim et al., 2019), while the other seven were reviews (Cao and Dixon, 2016; Xie et al., 2016; Gaschler and Stockwell, 2017; Stockwell et al., 2017; Fricker et al., 2018; Dodson et al., 2019; Bock and Tait, 2020). These papers contributed to the foundation of ferroptosis research in the field of the brain and help researchers understand the basics of this field.

The involvement of ferroptosis in many diseases has been proven (Tang et al., 2021). Especially in recent years, its targetable role in neurological diseases involving neurodegeneration, ischemic lesions and neurol tumors has been gradually revealed (Ashraf et al., 2020; Chen et al., 2020; David et al., 2021; Miao et al., 2022; Peeples and Genaro-Mattos, 2022). Furthermore, the inhibition of ferroptosis presented protective effects in models of cerebral hemorrhage in mice (Li et al., 2017). Iron dysregulation, lipid peroxidation, and mitochondrial abnormalities have been observed in patients with Alzheimer’s disease (AD) (Peña-Bautista et al., 2018). Additionally, iron dysregulation induced the decline of glutathione (GSH) and glutathione peroxidase 4 (GPX4) and ROS accumulation, and these factors together caused changes in AD markers such as amyloid beta peptide and Tau protein (Gleason and Bush, 2021). A close relationship between ferroptosis and Parkinson’s disease was established based on elevated iron levels in the brain (Jiang et al., 2017). Evidence shows that iron chelator treatment alleviated the motor damage of PD patients (Devos et al., 2014) and protected the blood-brain barrier (BBB) of PD model mice (Bar-Am et al., 2015). In addition, ferroptosis occurred in neuronal cells of stroke patients (Liu et al., 2022), and ferroptosis inhibitors reduced the neuronal damage of stroke patients (Alim et al., 2019). By inducing ferroptosis, the treatment effects of glioblastoma were improved in patients with drug resistance (Yakubov et al., 2021). These studies suggest a great prospect for targeting ferroptosis to treat the structural and functional abnormalities of the brain.

The following limitations of this study should not be ignored. First, we searched WOSCC (the most applied database in bibliometric studies) for relevant publications, so any publications excluded from WOSCC were missed. Second, we limited the timeline up to 31 December 2021; hence, any updated studies published during the writing and submission process of this article were also excluded from the analysis. Third, the drawback of VOSviewer and CiteSpace may limit the results of the current bibliometric analysis.

## Conclusion

In summary, this study reported the development tendency of ferroptosis research on the brain.

The results indicate that the recent global research on ferroptosis in the field of brain science is exploding. The most productive countries were China, the United States, and Australia. With the development of ferroptosis research in the brain, many keywords related to its mechanisms occurred with high frequency. “Ferroptosis” was the most cited keyword, followed by “cell death,” “oxidative stress,” “lipid peroxidation” and “iron.” “Amyloid precursor protein” was the keyword with the strongest bursts, while “neurodegenerative disease,” “iron homeostasis,” and “reactive oxygen species” had bursts until 2021. These keywords revealed the hotspots and frontiers of ferroptosis research in the brain science field. This research landscape analysis of ferroptosis in the brain will offer a new reference to build on future research.

## Data Availability

The original contributions presented in this study are included in the article/Supplementary Material; further inquiries can be directed to the corresponding authors.

## References

[B1] Alim I. Caulfield J. T. Chen Y. Swarup V. Geschwind D. H. Ivanova E. (2019). Selenium drives a transcriptional adaptive program to block ferroptosis and treat stroke. Cell 177 (5), 1262e25–1279. 10.1016/j.cell.2019.03.032 31056284

[B2] Ashraf A. Jeandriens J. Parkes H. G. So P. W. (2020). Iron dyshomeostasis, lipid peroxidation and perturbed expression of cystine/glutamate antiporter in Alzheimer's disease: Evidence of ferroptosis. Redox Biol. 32, 101494. 10.1016/j.redox.2020.101494 32199332 PMC7083890

[B3] Bar-Am O. Amit T. Kupershmidt L. Aluf Y. Mechlovich D. Kabha H. (2015). Neuroprotective and neurorestorative activities of a novel iron chelator-brain selective monoamine oxidase-A/monoamine oxidase-B inhibitor in animal models of Parkinson's disease and aging. Neurobiol. Aging 36 (3), 1529–1542. 10.1016/j.neurobiolaging.2014.10.026 25499799

[B4] Bock F. J. Tait S. W. G. (2020). Mitochondria as multifaceted regulators of cell death. Nat. Rev. Mol. Cell Biol. 21 (2), 85–100. 10.1038/s41580-019-0173-8 31636403

[B5] Cao J. Y. Dixon S. J. (2016). Mechanisms of ferroptosis. Cell. Mol. Life Sci. 73 (11-12), 2195–2209. 10.1007/s00018-016-2194-1 27048822 PMC4887533

[B6] Chen C. (2004). Searching for intellectual turning points: Progressive knowledge domain visualization. Proc. Natl. Acad. Sci. U. S. A. 101 (1), 5303–5310. 10.1073/pnas.0307513100 14724295 PMC387312

[B7] Chen S. Chen Y. Zhang Y. Kuang X. Liu Y. Guo M. (2020). Iron metabolism and ferroptosis in epilepsy. Front. Neurosci. 14, 601193. 10.3389/fnins.2020.601193 33424539 PMC7793792

[B8] Chen Y. Long T. Xu Q. Zhang C. (2021). Bibliometric analysis of ferroptosis in stroke from 2013 to 2021. Front. Pharmacol. 12, 817364. 10.3389/fphar.2021.817364 35264947 PMC8899397

[B9] David S. Jhelum P. Ryan F. Jeong S. Y. Kroner A. (2021). Dysregulation of iron homeostasis in the CNS and the role of ferroptosis in neurodegenerative disorders. Antioxid. Redox Signal. 10.1089/ars.2021.0218 34569265

[B10] Devos D. Moreau C. Devedjian J. C. Kluza J. Petrault M. Laloux C. (2014). Targeting chelatable iron as a therapeutic modality in Parkinson's disease. Antioxid. Redox Signal. 21 (2), 195–210. 10.1089/ars.2013.5593 24251381 PMC4060813

[B11] Dixon S. J. Lemberg K. M. Lamprecht M. R. Skouta R. Zaitsev E. M. Gleason C. E. (2012). Ferroptosis: An iron-dependent form of nonapoptotic cell death. Cell 149 (5), 1060–1072. 10.1016/j.cell.2012.03.042 22632970 PMC3367386

[B12] Do Van B. Gouel F. Jonneaux A. Timmerman K. Gelé P. Pétrault M. (2016). Ferroptosis, a newly characterized form of cell death in Parkinson's disease that is regulated by PKC. Neurobiol. Dis. 94, 169–178. 10.1016/j.nbd.2016.05.011 27189756

[B13] Dodson M. Castro-Portuguez R. Zhang D. D. (2019). NRF2 plays a critical role in mitigating lipid peroxidation and ferroptosis. Redox Biol. 23, 101107. 10.1016/j.redox.2019.101107 30692038 PMC6859567

[B14] Dong X. Tan Y. Zhuang D. Hu T. Zhao M. (2022). Global characteristics and trends in research on ferroptosis: A data-driven bibliometric study. Oxid. Med. Cell. Longev. 2022, 8661864. 10.1155/2022/8661864 35087622 PMC8787456

[B15] Fricker M. Tolkovsky A. M. Borutaite V. Coleman M. Brown G. C. (2018). Neuronal cell death. Physiol. Rev. 98 (2), 813–880. 10.1152/physrev.00011.2017 29488822 PMC5966715

[B16] Friedmann Angeli J. P. Schneider M. Proneth B. Tyurina Y. Y. Tyurin V. A. Hammond V. J. (2014). Inactivation of the ferroptosis regulator Gpx4 triggers acute renal failure in mice. Nat. Cell Biol. 16 (12), 1180–1191. 10.1038/ncb3064 25402683 PMC4894846

[B17] Gaschler M. M. Stockwell B. R. (2017). Lipid peroxidation in cell death. Biochem. Biophys. Res. Commun. 482 (3), 419–425. 10.1016/j.bbrc.2016.10.086 28212725 PMC5319403

[B18] Gleason A. Bush A. I. (2021). Iron and ferroptosis as therapeutic targets in alzheimer's disease. Neurotherapeutics 18 (1), 252–264. 10.1007/s13311-020-00954-y 33111259 PMC8116360

[B19] Jiang H. Wang J. Rogers J. Xie J. (2017). Brain iron metabolism dysfunction in Parkinson's disease. Mol. Neurobiol. 54 (4), 3078–3101. 10.1007/s12035-016-9879-1 27039308

[B20] Jiang X. Stockwell B. R. Conrad M. (2021). Ferroptosis: Mechanisms, biology and role in disease. Nat. Rev. Mol. Cell Biol. 22 (4), 266–282. 10.1038/s41580-020-00324-8 33495651 PMC8142022

[B21] Li G. Liang Y. Yang H. Zhang W. Xie T. (2022). The research landscape of ferroptosis in cancer: A bibliometric analysis. Front. Cell Dev. Biol. 10, 841724. 10.3389/fcell.2022.841724 35693942 PMC9174675

[B22] Li J. Cao F. Yin H.-L. Huang Z.-J. Lin Z.-T. Mao N. (2020). Ferroptosis: Past, present and future. Cell Death Dis. 11 (2), 88. 10.1038/s41419-020-2298-2 32015325 PMC6997353

[B23] Li Q. Han X. Lan X. Gao Y. Wan J. Durham F. (2017). Inhibition of neuronal ferroptosis protects hemorrhagic brain. JCI Insight 2 (7), e90777. 10.1172/jci.insight.90777 28405617 PMC5374066

[B24] Liu J. Guo Z. N. Yan X. L. Huang S. Ren J. X. Luo Y. (2020). Crosstalk between autophagy and ferroptosis and its putative role in ischemic stroke. Front. Cell. Neurosci. 14, 577403. 10.3389/fncel.2020.577403 33132849 PMC7566169

[B25] Liu Y. Fang Y. Zhang Z. Luo Y. Zhang A. Lenahan C. (2022). Ferroptosis: An emerging therapeutic target in stroke. J. Neurochem. 160 (1), 64–73. 10.1111/jnc.15351 33733478

[B26] Miao Z. Tian W. Ye Y. Gu W. Bao Z. Xu L. (2022). Hsp90 induces Acsl4-dependent glioma ferroptosis via dephosphorylating Ser637 at Drp1. Cell Death Dis. 13 (6), 548. 10.1038/s41419-022-04997-1 35697672 PMC9192632

[B27] Peeples E. S. Genaro-Mattos T. C. (2022). Ferroptosis: A promising therapeutic target for neonatal hypoxic-ischemic brain injury. Int. J. Mol. Sci. 23 (13), 7420. 10.3390/ijms23137420 35806425 PMC9267109

[B28] Peña-Bautista C. Vigor C. Galano J. M. Oger C. Durand T. Ferrer I. (2018). Plasma lipid peroxidation biomarkers for early and non-invasive Alzheimer Disease detection. Free Radic. Biol. Med. 124, 388–394. 10.1016/j.freeradbiomed.2018.06.038 29969716

[B29] Shen Z. Liu T. Li Y. Lau J. Yang Z. Fan W. (2018). Fenton-reaction-acceleratable magnetic nanoparticles for ferroptosis therapy of orthotopic brain tumors. ACS Nano 12 (11), 11355–11365. 10.1021/acsnano.8b06201 30375848

[B30] Stockwell B. R. (2022). Ferroptosis turns 10: Emerging mechanisms, physiological functions, and therapeutic applications. Cell 185 (14), 2401–2421. 10.1016/j.cell.2022.06.003 35803244 PMC9273022

[B31] Stockwell B. R. Friedmann Angeli J. P. Bayir H. Bush A. I. Conrad M. Dixon S. J. (2017). Ferroptosis: A regulated cell death nexus linking metabolism, redox biology, and disease. Cell 171 (2), 273–285. 10.1016/j.cell.2017.09.021 28985560 PMC5685180

[B32] Tang D. Chen X. Kang R. Kroemer G. (2021). Ferroptosis: Molecular mechanisms and health implications. Cell Res. 31 (2), 107–125. 10.1038/s41422-020-00441-1 33268902 PMC8026611

[B33] Tuo Q. Z. Lei P. Jackman K. A. Li X. L. Xiong H. Li X. L. (2017). Tau-mediated iron export prevents ferroptotic damage after ischemic stroke. Mol. Psychiatry 22 (11), 1520–1530. 10.1038/mp.2017.171 28886009

[B34] Viswanathan V. S. Ryan M. J. Dhruv H. D. Gill S. Eichhoff O. M. Seashore-Ludlow B. (2017). Dependency of a therapy-resistant state of cancer cells on a lipid peroxidase pathway. Nature 547 (7664), 453–457. 10.1038/nature23007 28678785 PMC5667900

[B35] Wenzel S. E. Tyurina Y. Y. Zhao J. St Croix C. M. Dar H. H. Mao G. (2017). PEBP1 wardens ferroptosis by enabling lipoxygenase generation of lipid death signals. Cell 171 (3), 628e26–641. 10.1016/j.cell.2017.09.044 29053969 PMC5683852

[B36] Wu H. Wang Y. Tong L. Yan H. Sun Z. (2021). Global research trends of ferroptosis: A rapidly evolving field with enormous potential. Front. Cell Dev. Biol. 9, 646311. 10.3389/fcell.2021.646311 33996807 PMC8116802

[B37] Xie Y. Hou W. Song X. Yu Y. Huang J. Sun X. (2016). Ferroptosis: Process and function. Cell Death Differ. 23 (3), 369–379. 10.1038/cdd.2015.158 26794443 PMC5072448

[B38] Xiong J. Qi W. Liu J. Zhang Z. Wang Z. Bao J. (2021). Research progress of ferroptosis: A bibliometrics and visual analysis study. J. Healthc. Eng. 2021, 2178281. 10.1155/2021/2178281 34413966 PMC8370827

[B39] Yakubov E. Eibl T. Hammer A. Holtmannspotter M. Savaskan N. Steiner H. H. (2021). Therapeutic potential of selenium in glioblastoma. Front. Neurosci. 15, 666679. 10.3389/fnins.2021.666679 34121995 PMC8194316

[B40] Yang L. Cao L.-m. Zhang X.-j. Chu B. (2022). Targeting ferroptosis as a vulnerability in pulmonary diseases. Cell Death Dis. 13 (7), 649. 10.1038/s41419-022-05070-7 35882850 PMC9315842

[B41] Zhang J. Song L. Xu L. Fan Y. Wang T. Tian W. (2021). Knowledge domain and emerging trends in ferroptosis research: A bibliometric and knowledge-map analysis. Front. Oncol. 11, 686726. 10.3389/fonc.2021.686726 34150654 PMC8209495

[B42] Zhao L. Zhou X. Xie F. Zhang L. Yan H. Huang J. (2022). Ferroptosis in cancer and cancer immunotherapy. Cancer Commun. 42 (2), 88–116. 10.1002/cac2.12250 PMC8822596 35133083

[B43] Zhou Q. Wu F. Zhao M. Yang M. (2021). Bibliometric evaluation of 2012-2020 publications on ferroptosis in cancer treatment. Front. Cell Dev. Biol. 9, 793347. 10.3389/fcell.2021.793347 35118077 PMC8804294

[B44] Zille M. Karuppagounder S. S. Chen Y. Gough P. J. Bertin J. Finger J. (2017). Neuronal death after hemorrhagic stroke *in vitro* and *in vivo* shares features of ferroptosis and necroptosis. Stroke 48 (4), 1033–1043. 10.1161/strokeaha.116.015609 28250197 PMC5613764

