# Supplementary material for: The research landscape of ferroptosis in the brain: A bibliometric analysis
Source: Front Pharmacol. 2022 Oct 18;13:1014550. doi: 10.3389/fphar.2022.1014550 (PMC9622939; doi:10.3389/fphar.2022.1014550)
Supplement: Supplementary file 3 [file Table1.DOCX]

Table 1. Top 10 most productive countries.

| Rank | Country | Documents | Citations | Total link strength |
| --- | --- | --- | --- | --- |
| 1 | China | 338 | 11846 | 101 |
| 2 | United States | 166 | 18038 | 134 |
| 3 | Germany | 64 | 5371 | 85 |
| 4 | Australia | 42 | 4926 | 57 |
| 5 | Japan | 33 | 2953 | 24 |
| 6 | France | 27 | 1890 | 52 |
| 7 | England | 24 | 1495 | 53 |
| 8 | Russia | 22 | 1162 | 41 |
| 9 | Italy | 17 | 623 | 8 |
| 10 | Canada | 15 | 574 | 21 |

Table 2. Top 10 most productive institutions.

|  | Organizations | Countries | Documents | Citations | Total link strength |
| --- | --- | --- | --- | --- | --- |
| 1 | The University of Melbourne | Australia | 34 | 4211 | 100 |
| 2 | University of Pittsburgh | United States | 23 | 4535 | 104 |
| 3 | Central South University | China | 18 | 409 | 18 |
| 4 | Zhejiang University | China | 18 | 513 | 33 |
| 5 | Helmholtz Zentrum Munchen | Germany | 15 | 1083 | 42 |
| 6 | Sichuan University | China | 15 | 723 | 21 |
| 7 | Salk Institute for Biological Studies | United States | 14 | 481 | 15 |
| 8 | Shanghai Jiaotong University | China | 13 | 400 | 22 |
| 9 | Soochow University | China | 12 | 315 | 19 |
| 10 | Sun Yat-Sen University | China | 12 | 150 | 6 |

Table 3. Top 10 authors and co-cited authors in field of ferroptosis in brain.

| Rank | Author | Count | H-index | Co-cited author | Count | H-index |
| --- | --- | --- | --- | --- | --- | --- |
| 1 | Ashley I. Bush | 20 | 112 | Scott J Dixon | 471 | 31 |
| 2 | Marcus Conrad | 16 | 15 | Wan Seok Yang | 380 | 17 |
| 3 | Scott Ayton | 14 | 35 | J Pedro Friedmann Angeli | 306 | 35 |
| 4 | Pamela Maher | 13 | 56 | Sebastian Doll | 305 | 9 |
| 5 | Mao Xiaoyuan | 10 | 24 | Brent R Stockwell | 303 | 30 |
| 6 | Zhou Honghao | 10 | 46 | Gao, Minghui | 297 | 16 |
| 7 | Hulya Bayir | 9 | 35 | Scott Ayton | 176 | 35 |
| 8 | Carsten Culmsee | 9 | 57 | Valerian E Kagan | 173 | 83 |
| 9 | Wang Jian | 9 | 50 | Xie Yangchun | 172 | 22 |
| 10 | Yoko Hirata | 7 | 28 | Li Qian | 171 | 25 |

Table 4. Top 10 journals and co-cited journals in field of ferroptosis in brain.

| Rank | Journal | Count | JCR  (2021) | IF  (2021) | Cited journal | Count | JCR  (2021) | | IF  (2021) | |
| --- | --- | --- | --- | --- | --- | --- | --- | --- | --- | --- |
| 1 | Free radical biology and medicine | 24 | Q1 | 8.101 | Cell | 1880 | Q1 | | 66.85 | |
| 2 | Frontiers in neuroscience | 21 | Q2 | 5.152 | Journal of biological chemistry | 1461 | Q2 | | 5.486 | |
| 3 | Cell death disease | 16 | Q1 | 9.685 | Free radical biology and medicine | 1365 | Q1 | | 8.101 | |
| 4 | Redox biology | 16 | Q1 | 10.787 | Nature | 1345 | Q1 | | 69.504 | |
| 5 | Frontiers in cell and developmental biology | 13 | Q1/Q2 | 6.081 | Proceedings of the national academy of sciences of the United States of America | 1239 | Q1 | | 12.779 | |
| 6 | Frontiers in cellular neuroscience | 11 | Q1 | 6.147 | Cell death and differentiation | 909 | Q1 | | 12.067 | |
| 7 | Cell death and differentiation | 10 | Q1 | 12.067 | Journal of neurochemistry | 790 | Q2 | | 5.546 | |
| 8 | Oxidative medicine and cellular longevity | 10 | Q2 | 7.31 | Nature chemical biology | 736 | Q1 | | 11.174 | |
| 9 | Cells | 9 | Q2 | 7.666 | Redox biology | 718 | Q1 | | 10.787 | |
| 10 | Frontiers in pharmacology | 9 | Q1 | 5.988 | Plos one | 676 | Q2 | 3.752 | |  |

Table 5. Top10 most cited documents in field of ferroptosis in brain.

| Rank | Title | Journal | First author | Year | Type | Citation |
| --- | --- | --- | --- | --- | --- | --- |
| 1 | Ferroptosis: An Iron-Dependent Form of Nonapoptotic Cell Death | Cell | Scott J Dixon | 2012 | Article | 4283 |
| 2 | Ferroptosis: A Regulated Cell Death Nexus Linking Metabolism, Redox Biology, and Disease | Cell | Brent R Stockwell | 2017 | Review | 2118 |
| 3 | Ferroptosis: process and function | Cell death and differentiation | Xie Yangchun | 2016 | Review | 1228 |
| 4 | Lipid peroxidation in cell death | Biochemical and biophysical research comminications | Michael M. Gaschler | 2017 | Review | 678 |
| 5 | Dependency of a therapy-resistant state of cancer cells on a lipid peroxidase pathway | Nature | Vasanthi S. Viswanathan | 2017 | Article | 633 |
| 6 | Mechanisms of ferroptosis | Cellular and Molecular Life Sciences | Cao Jennifer Yinuo | 2016 | Review | 578 |
| 7 | Mitochondria as multifaceted regulators of cell death | Nature reviews molecular cell biology | Florian J.  Bock | 2020 | Review | 518 |
| 8 | Neuronal cell death | Molecullar Pstchiatry | Michael Fricker | 2018 | Review | 394 |
| 9 | NRF2 plays a critical role in mitigating lipid peroxidation and ferroptosis | Redox biology | Matthew Dodson | 2019 | Review | 356 |
| 10 | PEBP1 Wardens Ferroptosis by Enabling Lipoxygenase Generation of Lipid Death Signals | Cell | Sally E  Wenzel | 2017 | Article | 339 |

Table 6. Top 20 keywords related to ferroptosis in brain.

| Rank | Keyword | Occurrence | Rank | Keyword | Occurrence |
| --- | --- | --- | --- | --- | --- |
| 1 | ferroptosis | 437 | **11** | mechanisms | 69 |
| 2 | cell death | 247 | **12** | activation | 67 |
| 3 | oxidative stress | 245 | **13** | death | 66 |
| 4 | lipid peroxidation | 198 | **14** | brain | 61 |
| 5 | iron | 163 | **15** | expression | 57 |
| 6 | glutathione peroxidase 4 | 108 | **16** | autophagy | 50 |
| 7 | metabolism | 92 | **17** | glutathione | 48 |
| 8 | parkinson's disease | 91 | **18** | inflammation | 48 |
| 9 | apoptosis | 82 | **19** | neurodegeneration | 48 |
| 10 | alzheimer's disease | 74 | **20** | cancer | 47 |
